# Rapid and sustained response to immune checkpoint inhibition in cutaneous squamous cell carcinoma after allogenic hematopoietic cell transplant for sézary syndrome

**DOI:** 10.1186/s40425-019-0801-z

**Published:** 2019-12-04

**Authors:** Karam Khaddour, Amy Musiek, Lynn A. Cornelius, Farrokh Dehdashti, Peter Westervelt, Ryan Fields, George Ansstas

**Affiliations:** 1 Division of Medical Oncology, Department of Medicine, Washington University in Saint Louis, 660 South Euclid Avenue, Campus Box 8056, St. Louis, MO 63110 USA; 2 Division of Medical Oncology, Bone Marrow Transplantation and Leukemia, Department of Medicine, Washington University in Saint Louis, St. Louis, USA; 3 Division of Dermatology, Department of Medicine, Washington University in Saint Louis, St. Louis, USA; 4 Alvin J. Siteman Cancer Center, St. Louis, USA; 5 Division of Nuclear Medicine, Department of Radiology, Washington University in Saint Louis, St. Louis, USA; 6 Section of Surgical Oncology, Department of Surgery, Washington University in Saint Louis, St. Louis, USA

**Keywords:** Cutaneous squamous cell carcinoma, Cutaneous T-cell lymphoma, Sézary syndrome, Immune checkpoint inhibitors, Allogenic hematopoietic cell transplant, Pembrolizumab, Graft versus host disease

## Abstract

**Background:**

Cutaneous squamous cell carcinoma (cSCC) is not uncommon in association with indolent malignancies that were treated with prior radiotherapy and after allogenic bone marrow transplantation. On the other hand, cutaneous T-cell lymphoma (CTCL) is a subtype of non-Hodgkin’s lymphoma which is characterized by an indolent course, with relative refractoriness to conventional chemotherapies and radiotherapy, and occasionally referred for allogeneic hematopoietic cell transplantation (allo-HCT). Recently, the use of immune checkpoint inhibitors has gained attention in the treatment of both cutaneous squamous cell carcinoma and hematological malignancies. However, many patients with hematological malignancies eventually undergo allo-HCT, raising the concern of potential adverse events (graft versus host disease) due to manipulation of the immune system with use of checkpoint inhibitors.

**Case presentation:**

We describe a patient with relapsed refractory CTCL (Sézary Syndrome) who underwent allo-HCT with persistence of disease post-transplant. The patient additionally developed a progressively worsening lesion on the right shoulder which was biopsied and showed poorly differentiated carcinoma (cSCC). Pembrolizumab was started for the treatment of cSCC. After second cycle of treatment, the cSCC lesion responded dramatically to the use of immune checkpoint inhibitor. Also, the patient experienced significant resolution of pruritus and generalized erythema. During 24 months of follow up after initial treatment with checkpoint inhibition immunotherapy, the patient showed durable response of both cSCC and CTCL, as well as restoration of full donor chimerism, without obvious worsening of graft versus host disease (GVHD).

**Conclusion:**

This is the first case to our knowledge of rapid and durable response of both cSCC and CTCL to immune checkpoint inhibition after allo-HCT. Although this report highlights the potential for significant response to this class of medication, further studies are required to confirm the efficacy and safety of this approach in patients with CTCL after allo-HCT given the potential concern of GVHD.

## Background

Conventional chemotherapies are considered not to be curative in the majority of cutaneous T-cell lymphomas (CTCL) [1]. Recently, the use of immune checkpoint inhibitors has largely expanded to include hematological malignancies, specifically relapsed/refractory chemoresistant Hodgkin’s Lymphoma (r/r-cHL) and primary mediastinal large B-cell lymphoma [2, 3].The rationale relies on the fact that genetic alterations occurring in the microenvironment of lymphomas specifically in the programmed death ligand receptor (PD-L1/PD-L2) loci can lead to overexpression of PD-L1/2 on malignant cells, which helps tumor cells to evade the effective antitumor immune response [4]. Similarly, CTCL such as Mycosis Fungoides (MF) and Sézary Syndrome (SS) can debilitate the immune response against malignant cells in the tumor microenvironment and thus could be considered a target for therapies that can restore immune surveillance [5]. However, there is a concern for the use of immune checkpoint inhibitors in patients with lymphoma who undergo allogenic hematopoietic cell transplantation due to the possibility of triggering or aggravating graft versus host disease. This has led to lack of literature regarding the safety and efficacy of immune checkpoint inhibitors in this population as patients with prior history of allo-HCT were excluded from clinical trials which examined the efficacy of immunotherapy. Moreover, advanced cutaneous squamous cell carcinoma has been shown to have a high mutational burden which could increase the expression of tumor neoantigens [6]. Also, the association with PD-L1 expression is established with cutaneous squamous cell carcinoma which prompted the study of immune checkpoint inhibitors as a potential therapy [7].

We describe the first case to our knowledge of a patient with a history of allo-HCT who had a rapid and durable response of both cSCC and CTCL/SS after treatment with immune checkpoint inhibitors.

## Case presentation

**Fig. 1:**
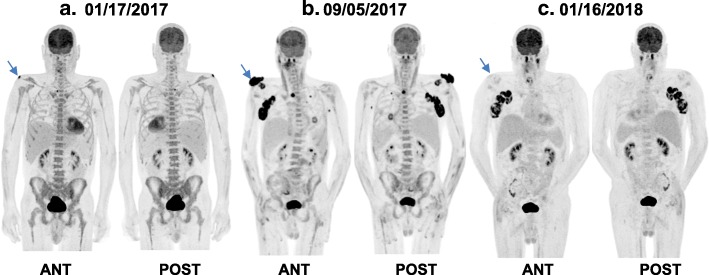
Anterior and posterior volume-rendered maximum activity-reprojection FDG-PET images showing (**a**) FDG uptake in the right shoulder (arrow) and a paratracheal lymph node. **b** markedly hypermetabolic lesion in the right shoulder with SUV_max_ of 26.7 (arrow), markedly hypermetabolic right axillary and right paratracheal lymphadenopathy with SUV_max_ of 27.3 with interval development of hypermetabolic focal cutaneous lesions, left axillary lymph node and pulmonary lesions. **c** near complete response of the right shoulder hypermetabolic lesion (arrow), complete resolution of paratracheal lymph node, left axillary lymph node, pulmonary and cutaneous lesions, with persistent FDG uptake of the right axillary lymph nodes

**Fig. 2:**
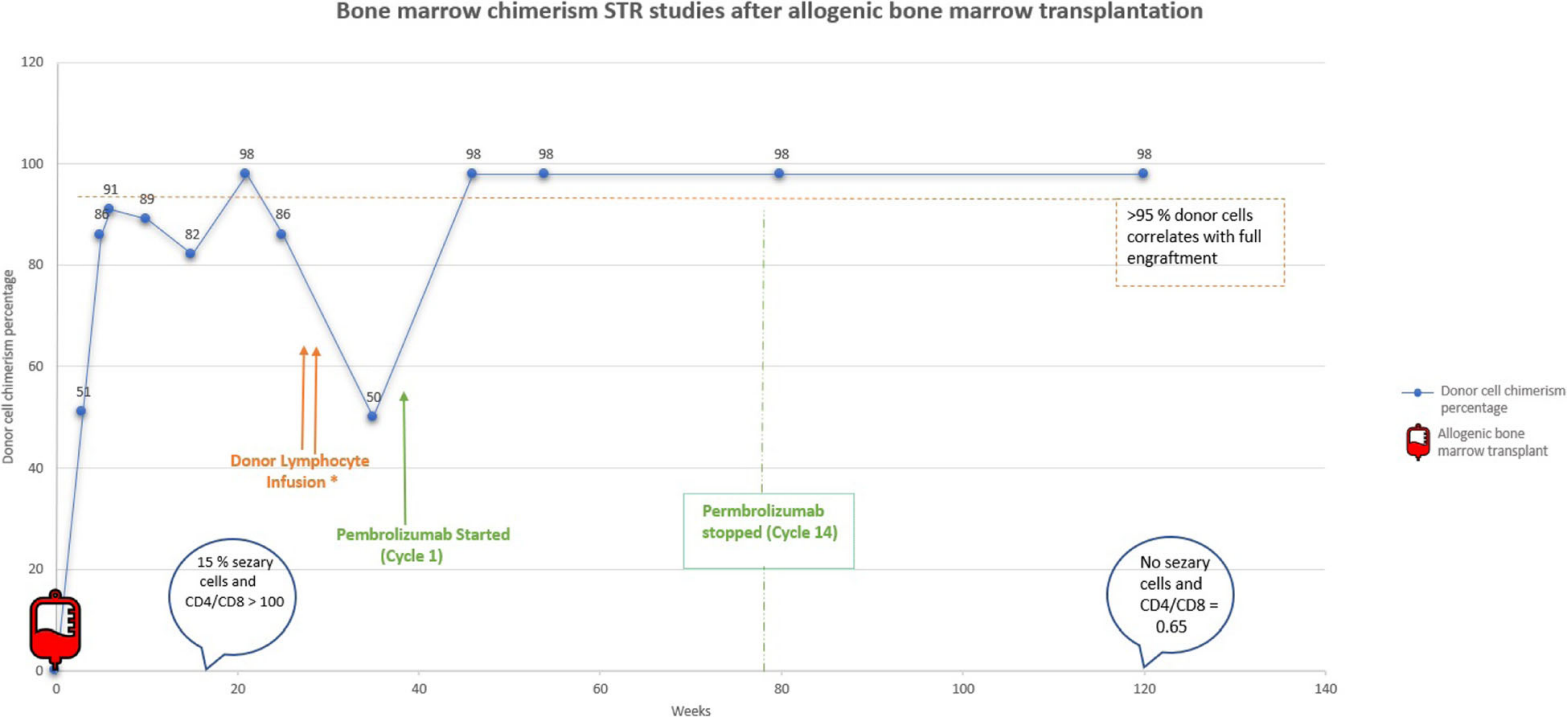
Timeline of bone marrow chimerism performed with STR studies after bone marrow transplantation. * Donor lymphocyte infusion was administered on two separate doses on weeks 26 and 31. Pembrolizumab cycle 1 was at week 36 and last cycle (cycle 14) was at week 78

**Fig. 3:**
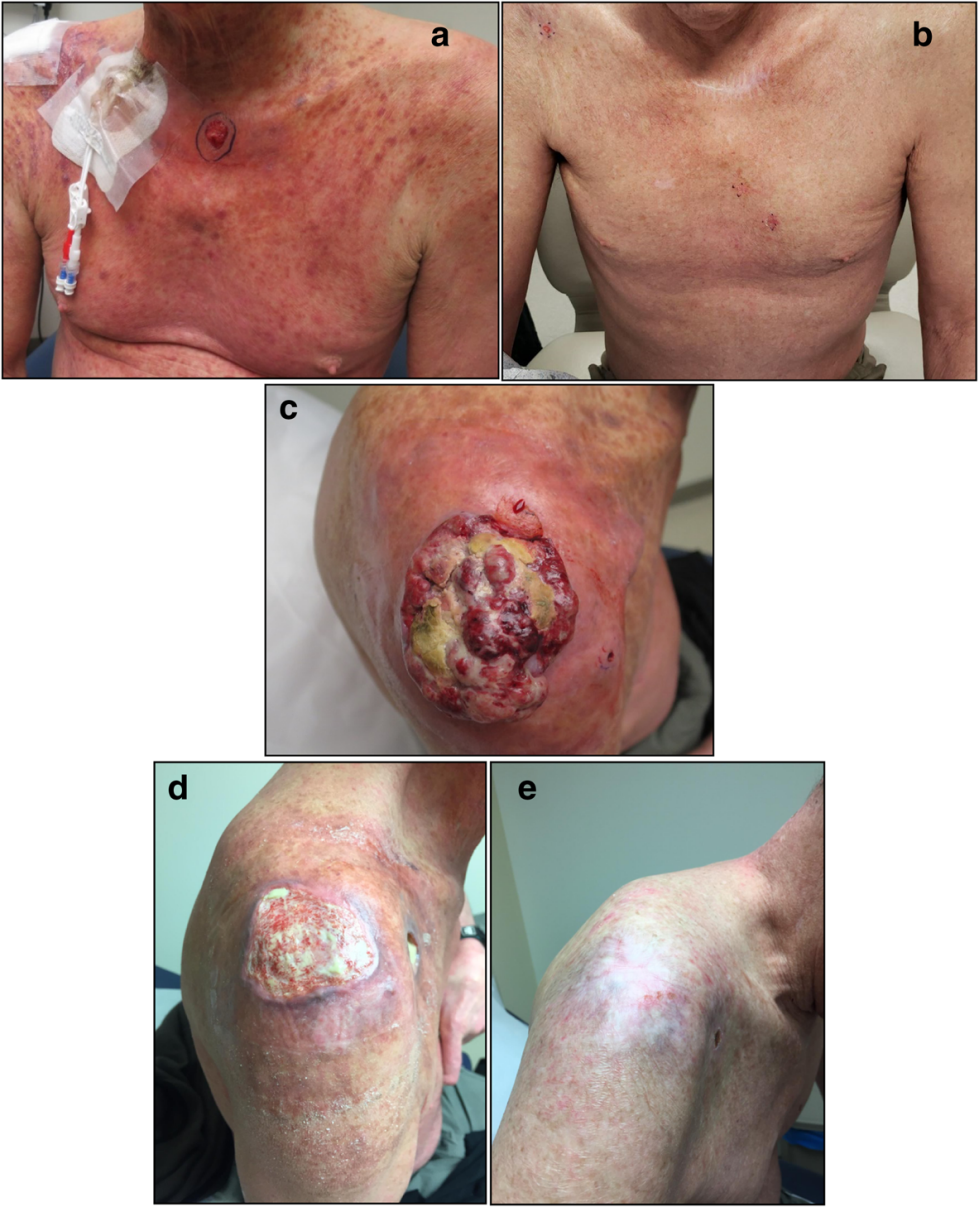
Skin lesions of CTCL and cSCC before and after PD-1 inhibition. **a**: Generalized eruption of small confluent erythematous macules and papules on the anterior chest wall after stem cell transplant and before pembrolizumab. **b**: resolution of the previously mentioned erythematous maculopapular eruption after pembrolizumab. **c**: multiple firm nodules with partial ulceration and keratin deposition on the superior aspect of the right shoulder representing poorly differentiated squamous cell carcinoma (before pembrolizumab), also erythematous eruption can be noted around the cSCC lesion which represents cutaneous lymphoma. **d** & **e**: Granular tissue with skin regeneration replacing the nodular ulcerated lesions of cSCC (after pembrolizumab), there is complete resolution of the papular erythematous rash. *CTCL: cutaneous T cell lymphoma, cSCC: cutaneous squamous cell carcinoma*

A 58-year-old Caucasian male with relapsed/refractory Sézary Syndrome (r/r SS) stage IVA was referred for allogeneic hematopoietic cell transplantation (allo-HCT). The patient had received multiple therapies prior to being assessed for a transplant (Table 1). Positron Emission Tomography/Computed Tomography (PET/CT) with [^18^F]fluorodeoxyglucose (FDG) prior to transplant (1/17/2017) revealed interval development of a new hypermetabolic soft tissue nodule in the skin of the posterior right shoulder with maximum standard uptake value (SUV_max_) of 12.3 and a hypermetabolic right paratracheal lymph node (Fig. 1). Skin biopsy was consistent with residual CTCL/SS, a bone marrow biopsy demonstrated hypocellularity with extensive involvement of mature T-cell lymphoma (79% by flow cytometry), and cytogenetic studies revealed no aberrations. The patient underwent allogeneic hematopoietic cell transplantation (allo-HCT) using a fully matched male sibling donor. The myeloablative conditioning regimen prior to transplant consisted of hyperfractionated total body irradiation combined with high dose cyclophosphamide. Graft versus host disease (GVHD) prophylaxis consisted of tacrolimus and methotrexate. Tacrolimus was subsequently changed to sirolimus due to peri-transplant neurotoxicity. Shortly after transplantation (week 7), the patient developed a diffuse erythematous rash, a biopsy of which was most consistent with skin GVHD (vs CTCL vs drug rash) and received prednisone, mycophenolate mofetil (MMF) with ongoing sirolimus with improvement, and subsequent tapering to low dose prednisone (5 mg daily/ week 23). A bone marrow biopsy 4 months after allo-HCT showed persistent marrow (and peripheral blood) involvement with sézary cells (15% bone marrow involvement by mature T-cell lymphoma with CD4+/CD8+ ratio > 100). There was also mixed chimerism by short tandem repeat (STR) assay with 82% donor cells (Fig. 2). There was incomplete resolution of the skin manifestations of CTCL, which included generalized erythema and pruritus. Repeated skin biopsies were consistent with residual CTCL/SS. Five months after allo-HCT, STR studies again demonstrated persistent mixed chimerism, and donor lymphocyte infusions (DLI) in two separate doses were administered on weeks 26 and 31 (1 and 3 X 10e7 CD3 cells/kg, respectively) without complete resolution of skin symptoms or erythema. Following allo-HCT, there was a gradual worsening of a right shoulder skin lesion noted on PET/CT prior to transplant, and thought to be related to mycosis fungoides secondary to CTCL. The skin mass eventually extended over the superior aspect of the right shoulder with ulceration and hard induration, and 3 months post allo-HCT, the patient underwent subsequent involved-field radiotherapy with a total dose of 50 Gy. The lesion persisted and grew to 17 × 10 cm in maximum dimension with worsening necrosis, ulceration, and spread to the anterior chest wall (Fig. 3), a skin biopsy was performed and histopathology revealed poorly differentiated squamous cell carcinoma. Magnetic Resonance Imaging (MRI) of the shoulder showed a right lesion with no bone or muscular invasion, but there was extensive right axillary lymphadenopathy. FDG-PET/CT revealed worsening of disease with extensive markedly hypermetabolic soft tissue thickening in the right shoulder with SUV_max_ of 26.7 and interval development of markedly hypermetabolic partially necrotic right axillary lymphadenopathy with SUV_max_ of 27.3, as well as worsening in FDG uptake of the right paratracheal lymph node. There was interval development of hypermetabolic focal cutaneous lesions, left axillary lymph nodes and pulmonary lesions (T3N2B; Stage IV SCC) (Fig. 1).

**Table 1:** Sequence of systemic therapies administered since diagnosis of cutaneous T-cell lymphoma/ Sézary Syndrome

Prior therapy for CTCL/SS	Sequence and Duration	
Photochemotherapy (PUVA)	First Line of Therapy	8 months (2009)
Photopheresis	Second Line of Therapy	31 months (2009–2012)
Romidepsin	Third Line of Therapy	48 months (2010–2014)
Pegylated liposomal doxorubicin	Fourth Line of Therapy	3 months (2014)
Gemcitabine	Fifth Line of Therapy	1 month (2014)
Alemtuzumab	Sixth Line of Therapy	2 months (2015)
Mogamulizumab	Seventh Line of Therapy	1 month (2015)
Total skin electron beam therapy	Eighth Line of Therapy	3 months (2016)
Bexarotene	Ninth Line of Therapy	2 months (2016)
Pralatrexate	Tenth Line of Therapy	2 months (2016)
ICE (Ifosfamide, Carboplatin, Etoposide)	Eleventh Line of Therapy	3 months (2016–2017)
Sibling allogenic stem cell transplantation	Twelfth Line of Therapy	(2017)

After discussion of treatment options with the patient, intravenous pembrolizumab was started at a dose of 200 mg every 3 weeks. The patient had an Eastern Cooperative Oncology Group performance of (ECOG 3) prior to beginning of treatment. After 2^nd^ cycle of pembrolizumab, a macular rash appeared on the lower back. Skin biopsy demonstrated superficial perivascular mixed inflammatory cell infiltrate with normal CD4+/CD8+ ratio and was consistent with grade I skin immune related adverse event (IRAEs) due to pembrolizumab. Prednisone 60 mg was started and tapered over a total of 4 weeks with complete resolution of the skin symptoms secondary to IRAEs. The cutaneous manifestations including generalized erythema and pruritus which were secondary to CTCL resolved completely after the 2^nd^ cycle of immunotherapy. A FDG-PET/CT after the 5^th^ cycle of pembrolizumab showed marked metabolic response and near-complete resolution of the superficial right shoulder mass (SUV_max_ of 1.9), and complete resolution of right paratracheal and left axillary lymph nodes, as well as pulmonary and cutaneous lesions. There was decreased but persistent FDG uptake in the necrotic right axillary lymph nodes (SUV_max_ of 18.2) (Fig. 1). Given the rapid and significant response, pembrolizumab was continued for another three cycles and repeated PET/CT showed continued response with decreased FDG avidity in the right axilla. After cycle 14, the patient underwent right axillary lymph node dissection encompassing levels 1–3 nodes for assessment of the residual persistent lymphadenopathy on the right side. Histopathology demonstrated metastatic squamous cell carcinoma in 3 of 17 lymph nodes. His post-operative course was uncomplicated. The complete course of pembrolizumab treatment consisted of 14 cycles, over a course of 9 months. Follow up 24 months after initiation of immune checkpoint blockade therapy (14 months after stopping pembrolizumab) revealed no evidence of recurrence of cSCC. There was no compelling evidence of either progression of CTCL/SS or a flare in GVHD throughout his course of checkpoint inhibition therapy (most recent peripheral blood flow cytometry on week 120 showing no morphologic or immunophenotypic evidence of atypical lymphocytosis or sézary cells and CD4+/CD8+ ration of 0.65).

## Discussion

Systemic treatment of locally advanced and metastatic cutaneous squamous cell carcinoma has been limited with the exception of recent data supporting the potential role of immune checkpoint blockade specifically cemiplimab [8] among other ongoing trials with other PD-1 and PD-L1 inhibitors (Clinical Trials.gov. Identifiers: NCT02978625, NCT02964559, NCT03284424 and NCT03108131). Also, the data supporting the use of immune checkpoint inhibitors in the treatment of hematological malignancies other than classical Hodgkin’s Lymphoma (cHL) or primary mediastinal large B-cell lymphoma is limited. A new incentive of implementing immune checkpoint blockade in the treatment of T-cell lymphomas is supported by preclinical studies that showed programmed cell death ligand-1 (PD-L1) receptors to be expressed on malignant cells which contribute to suppression of host immunity against the malignant cells. This leads to overgrowth in T-cell clones derived from non-Hodgkin lymphoma [9]. Our case demonstrates a rapid and durable response after treatment with immune checkpoint inhibitors manifested by a resolution of CTCL/SS symptoms within 6 weeks and a remission period of 24 months since initiation of a PD-1 inhibitor. The indication to use pembrolizumab in our patient was for the treatment of poorly differentiated cutaneous squamous cell carcinoma. The theoretical possibility of a derived benefit against CTCL/SS was taken into consideration when starting treatment. Also, the possibility of exacerbating GVHD was also considered. Both poorly differentiated cutaneous squamous cell carcinoma (cSCC) and CTCL/SS responded rapidly within 6 weeks (2 cycles) after initiation of PD-1 inhibitor, without an evidence of GVHD flare.

Early phase clinical trials using the PD-1 inhibitor (nivolumab) in refractory/relapsed cutaneous T-cell lymphomas demonstrated different objective response rates (ORR) ranging from 15% in mycosis fungoides (MF) to 40% in peripheral T-cell lymphoma/Sézary Syndrome (SS) in phase I studies (number of evaluable patients = 18) [10]. However, the previous study excluded patients with prior allogenic hematopoietic cell transplant. A different study using pembrolizumab demonstrated an ORR of 38% in 24 evaluable patients with MF/SS stages Ib-IV with the longest duration of response reported to be 46 weeks [11]. Interestingly, there were higher response rates in retrospective studies examining the role of PD-1 inhibition after allo-HCT compared to patients who received immunotherapy prior to transplant for r/r-cHL [12, 13]. These two previous retrospective studies showed ORR of 95 and 77% with 1 –year progression free survival (PFS) of 85.2% compared to ORR of 75 and 87% respectively in patients who received immune checkpoint inhibitors prior to transplant [2, 12–14]. This raises the hypothesis that immune checkpoint blockade could have a synergistic role after allo-HCT or a role in homing of donor T-cells leading to an enhanced graft versus tumor effect. Some data support the previous hypothesis as immune escape is one of the mechanisms considered to cause relapse after transplant in hematological malignancies, and T-cell exhaustion is a potential mechanism of relapse after allo-HCT due to overexpression of PD-1/PD-L1 receptors, which leads to inactivation of effective T-cells in the tumor microenvironment [15]. Norde et al. found that relapsed myeloid leukemia after allo-HCT had higher expression and upregulation of PD-L1 receptors on progenitor malignant clones. It was also observed in the same study the suppressive effect on allogenic CD3+ T-cells when there was high expression of PD-L1 on malignant leukemic cells. Interestingly, blockade of the PD-1/PD-L1 interaction can augment the expansion of effector CD8+ T-cells and reactivate unresponsive T-memory cells in the microenvironment required for graft versus leukemia effect [15]. Moreover, there is a small body of evidence supporting a role of immune checkpoint inhibitors in the bone marrow microenvironment in acute myeloid leukemia (AML), myelodysplastic syndrome (MDS) and myelopthisis due to melanoma [16, 17].

The concern with the use of immune checkpoint inhibitors after allo-HCT is the development of graft versus host disease (GVHD). Conflicting data exist regarding the occurrence of this adverse event, with some studies reporting increased incidence and worsening of preexisting GVHD with 30% (6 of 20 patients), and 26% deaths in patients who received immune checkpoint blockade after allo-HCT [12, 13]. In the previous two retrospective studies there were 10 deaths related to GVHD in 51 patients (23 of whom developed GVHD) [12, 13]. However, another retrospective study assessing patients who were treated with immune checkpoint inhibitors after allo-HCT did not report GVHD development in any of the 7 evaluable patients [18].

The largest systematic review assessing the risk of GVHD with immune checkpoint inhibitors after allo-HCT showed that 49% of patients who developed GVHD had a prior history of the disease [19]. Some of the predictive factors of GVHD development in this patient population were higher doses of immune checkpoint inhibitors, shorter intervals between transplant and start of immunotherapy, and previous history of GVHD. This systematic review also supports other observations of higher response rates when immune checkpoint inhibitors were administered after transplant compared to those who received immunotherapy before they underwent allo-HCT [12, 13, 19].

In our case, we did not observe GVHD after treatment with PD-1 inhibitors. In fact, the patient’s erythema and pruritus which were manifestations of patient’s CTCL improved significantly after starting pembrolizumab. Moreover, there was a dermatological immune related adverse event observed in the patient during treatment (after 2nd cycle of pembrolizumab) which correlated with the response of both cSCC and CTCL. The previous observation is supported by a retrospective study showing robust immune response to PD-1/PD-L1 inhibitors when lichenoid and spongiotic IRAEs develop during treatment with PD-1/PD-L1 inhibitors [20].

It should be noted that donor lymphocyte infusion (DLI) which was performed in our patient on weeks 26 and 31 could have potentially led to full engraftment of allo-HCT resulting in the response observed in CTCL. The median response from administration of DLI to full engraftment is estimated to be 8–12 weeks [21]. However, the response of cSCC and CTCL in our case was observed to occur only after the administration of pembrolizumab (first cycle was at week 36) and the substantial deep response was observed after the second cycle which was at week 39.

In conclusion, our case is the first to describe a rapid and sustained clinical response of both CTCL/SS and cSCC to immune checkpoint inhibition after allo-HCT, without development of GVHD. However, this observation should be interpreted with caution given the non-trivial concern of GVHD. Further larger studies are needed to confirm the efficacy of immune checkpoint inhibitors and their safety profile in this patient population.

## Data Availability

Data was acquired through patient chart review using Washington University in Saint Louis medical records.

## Abbreviations

Allo-HCT: allogenic hematopoietic stem cell transplant
CTCL: Cutaneous T cell lymphoma
GVHD: Graft versus host disease
IRAEs: Immune related adverse events
PD-1: Programmed death receptor 1
PET/CT: Positron emission tomography- computed tomography
r/r-cHL: Relapsed/refractory classical Hodgkin’s lymphoma
cSCC: Cutaneous squamous cell carcinoma
SS: Sézary syndrome
